# Democracy’s limited impact on innovation: Panel data evidence from developing countries

**DOI:** 10.1371/journal.pone.0297915

**Published:** 2024-03-15

**Authors:** Masood Ahmed, Muhammad Atif Khan, Anam Attique, Muhammad Asif Khan, Hossam Haddad, Nidal Mahmoud Al-Ramahi

**Affiliations:** 1 Department of Public Administration, University of Kotli, Kotli, Azad Jammu and Kashmir, Pakistan; 2 Department of Commerce, University of Kotli, Kotli, Azad Jammu and Kashmir, Pakistan; 3 Department of Public Administration, MS Scholar, University of Kotli, Kotli, Azad Jammu and Kashmir, Pakistan; 4 Business Faculty, Zarqa University, Zarqa, Jordan; 5 Accounting Department, Zarqa University, Zarqa, Jordan; CNR: Consiglio Nazionale delle Ricerche, ITALY

## Abstract

This study investigates the relationship between democracy and innovation across 61 developing countries from 2013 to 2020, utilizing data from Global Innovation Index. Employing the Freedom House Index and Polity2 indicators as proxies for democracy, research employs Ordinary Least Squares (OLS), Fixed Effects and SystemGMM techniques to analyze their impact on innovation. The findings of the study reveal no statistically significant relationships between democracy and innovation in developing nations within specified timeframe. Through empirical analysis, including various econometric approaches, it is observed that the level of democracy as measured by these indicators, does not appear to exert a discernable impact on the innovation landscape of these countries. These results carry important implications for public policy. While the promotion of democracy remains a crucial goal, especially for societal development and political stability, this study suggests that solely focusing on enhancing democratic institutions might not necessarily yield immediate direct improvements in the innovation capacities of developing nations. Policymakers and stakeholders involved in fostering innovation ecosystems in these regions may need to consider a more nuanced approach, encompassing factors beyond the scope of democratic governance to effectively spur innovation. Understanding the nuanced relationship between democracy and innovation in developing countries has significant implications for designing targeted policies aimed at enhancing innovation capacities, economic growth and overall societal development in these regions.

## 1. Introduction

Innovation plays a pivotal role in driving economic performance and fostering growth in both developed and developing economies [1]. It is the catalyst that propels societies forward by introducing new ideas, technologies, products, and processes. Cai and Zhang [2] emphasized the importance of technological innovation for economic performance, leading to increased attention from scholars, firms, and governments.

The key drivers of science and technology production by nations are endless human wants, the goal of leadership in the presence of inter- and intra-group competition/conflict, and the control of nature. Individuals play a crucial role in the production of science advances and new technology. The individuals who are inspired, curious, and self-motivated, and who wish to learn and extend themselves, master new skills, and apply their talents responsibly. Individuals have a natural tendency to seek out novelty and challenges, to explore, to learn, and to achieve goals within efficient organizations in line with national interests. These factors guide scientific and technological advances within and between nations and generate socio-economic evolution and human progress in society. Furthermore, science advances and new technology are drivers of economic and productivity growth for nations and of a higher well-being of citizens [3].

Prior studies have concentrated on diverse determinants that impact innovation, including but not limited to research and development (R&D), economic growth, safeguarding of property rights, education, educational quality, and governmental policies [4,5]. Democratization has also been identified as a driving force for technological and economic change, leading to innovations that have reshaped industries and markets [6]. The causal relationship between democracy and innovation has received attention from scholars for example see Coccia [7], Coccia [8], Popper [9], Popper [10], Gao, Zang [11] and Wang, Feng [12]. Most of these studies establish a positive link between democracy and innovation.

Democratic societies prioritize the protection of individual freedoms and rights while establishing institutions that promote discoveries (inventions) in science and technology and safeguard the rights to intellectual property. In contrast to democratic nations, nondemocratic countries tend to prioritize collective action and robust state leadership to propel innovation and technical advancements. Karl Popper argued that democratic and liberal social structures are better at fostering innovation, while Kuhn [13] expressed skepticism about the significance of social and institutional factors in subversive innovation and the emergence of new scientific paradigms.

Scholars have conducted extensive research on the influence of a democratic system on economic development and development policies see for example Olson [14], Lipset [15], Barro [16], Rock [17] and Doucouliagos and Ulubaşoğlu [18]. This research has established correlations between democracy and growth, democracy and development, and democracy (culture or institutions) and developmental policies [19]. Academic literature has also acknowledged the correlation between developmental policies and innovation, along with the interrelatedness of growth, development, and innovation [20–22]. As a result, scholarly investigations have put forth a hypothesis regarding the correlation between democracy and innovation [23]. Notwithstanding the considerable theoretical and empirical literature, conclusive findings regarding the direct influence of democracy on innovation have yet to be established. The lack of long-term data has hindered the direct verification of Popper’s hypothesis.

This study aims to investigate the influence of democracy on innovation in developing countries. By drawing upon the tenets of political economy and partisan theory, it is widely posited that democracies advance individual liberties and safeguard property rights, thereby creating an enabling environment for developing and implementing novel technologies. According to Popper [9], Popper [10], democratic nations demonstrate superior innovation performance due to their developmental policies, protection of individual freedom, and safeguarding of property. However, empirical testing of Popper’s claims has been limited, with previous studies primarily focusing on the relationship between democracy and economic development, economic freedom, and property rights [23–26]. Some scholars have explored the connections between economic freedom and innovation [27] and intellectual property protection and innovation [28], suggesting potential links between democracy and innovation.

This study seeks to address several questions: Does democracy have an impact on innovation in developing countries? If so, which component of democracy has more influence on innovation, political rights, or civil liberties? Previous empirical tests examining the influence of democracy on innovation have reported positive relationship, except for Gao, Zang [11] which found no reliable influence. This study utilizes global innovation index data covering developing countries and suitable estimation methods such as fixed effects and the system generalized method of moment (GMM). It also incorporates previous innovation performance into the model to account for innovation progress and potential endogeneity.

The structure of the paper is as follows. After introduction follows the literature review section followed by data and methods section, results, discussion and finally conclusion section.

## 2. Literature review

History is a witness that whenever there is a need for innovation, a more accessible environment and attitude are needed. When we talk about promoting innovation within an organization, the top leadership and their policies play a very important role [29]. While we talk about innovation at the national level, the country’s politics and policies also play a vital role along with several other factors. Based on political economy and party theory, democracies mainly help in well-being, whether it is public well-being or overall national well-being, that sometimes directly or indirectly enhances innovation development within the country by starting such institutions that promote the use of updated and new technologies. As per studies, factors such as education, population, and the political environment can have much to do with the innovation process [30,31].

Subsequently, upon conducting a more comprehensive analysis of the factors mentioned above, it becomes apparent that governmental policies about education and finance also significantly influence the advancement of innovative practices. [12]. Specifically, the process of R&D expenditure is determined by the targeted policy of innovation activities, while economic development and education can be influenced by tax policy and education policy [32]. Along with this, democratic and authoritarian politics play essential but different roles while making various decisions for the government authorities [33].

Moreover, studies have also shown that as more accepting of true freedom, democratic regimes are reliable and show more accepting behavior towards freedom and exploring new ideas and innovation. Gerring, Bond [34] say that democratic politics favors creativity compared to autoerotic ideology as they are open to new challenges and bring more political stability. Apart from this, it has also been seen that if we talk about any country’s capital and resources, a democratic country is always rich in political capital. The same conclusion can be drawn by different studies that democratic government always promotes innovation by creating different forms of capital as protection of liberty and property rights. Sturm and de Haan [35] further elaborated that economic stability, a primary product of democratic government, also promotes creativity.

Sirowy and Inkeles [36] backed Sturm’s study by adding how democratic regimes give a boost to economic growth. In addition, de Haan and Sturm [37] stated that if rapid growth can bring dynamic elements to a specific position and make income independent of government and also if the political environment of any country is adopting liberal principles, then they are definitely the leading cause of the economic and financial development. On the contrary autocratic political environment of the country provides fewer opportunities for innovation on an economic and a human basis [18]. We move forward to analyze the relationship between economic freedom and innovation. In this regard, many studies show many factors are linked with innovation, and if a country or even an organization want to bring innovation in their workplaces they need to go beyond their limits as innovation is not possible in a vacuum space [38,39]. Florida [40], Florida [41] also argued the same thing in order to bring innovation, one needs to be creative with intelligence, and for this regard, new technologies and freedom to new experience are needed; this also means higher freedom fostering knowledge flows, advance technologies, diversity, and creativity. Stiglitz [42] stated that a peaceful environment, as well as stable and good economic circumstances, are needed for exposure to new technologies, while Gao, Zang [11] also stated that a natural and stress-free surrounding is also the basis for the promotion of individual initiatives, which is crucial for introducing new ideas, updated activities and revolution. Similarly, de Haan and Sturm [37] also noted that easy access to free markets is essential for economic development, and a focus on property rights protection can positively affect innovation through the upgradation of property rights protection and external market competition. The whole scenario clearly states that economic freedom provided by democratic systems improves productivity and leads towards a higher level of creativity and innovation.

As North [43] has argued, stability in political and civil rights provides an environment that helps in the protection of property rights, while erratic confiscation affects the whole situation negatively. In addition, Leblang [25] has demonstrated that democratic regimes tend to prioritize safeguarding private property rights, as opposed to autocratic regimes. Additionally, individual property rights have been found to correlate positively with market activity. This is because property rights provide individuals with greater freedom to engage in economic activities, thereby facilitating the production and exchange of goods. Olson [14] suggested that while the government is characterized by the protection of private property rights and the enforcement of contracts, people can conduct transactions for a long time and from which the country can also be benefited by getting profit and getting new investment opportunities.

Olson [14] analyzed the political condition of different countries and came to the conclusion that democratic countries protect property right better, and if property rights are protected and given accordingly, it greatly works for the economic development of democratic countries. The author’s statement highlights the correlation between enduring democracy and the necessity of individual property rights. Moreover, it suggests that democratic nations are more inclined to prioritize safeguarding individual rights and the implementation of contractual obligations. Compared to other nations, democratic regimes frequently exhibit a greater degree of safeguarding of private property. Similarly, De Haan and Siermann [44] argued that since property is at the core of material progress, democracies perform better in property protection.

According to scholars like Gao, Zang [11], intellectual property stock holds significant importance in driving national innovation. The safeguarding of property rights serves to stimulate the introduction and implementation of novel technologies by incentivizing innovators to engage in the realm of invention. [45,46]. Sweet and Eterovic Maggio [47] conducted an empirical analysis to examine the impact of intellectual property rights on innovation. The findings indicate a positive correlation between property rights and technological innovation. Democratic governments’ property rights provision to the general public offers defensive advantages that create a sense of security and tranquility. This, in turn, encourages investment in new technologies or products developed by individuals or institutions, thereby promoting technological progress. In comparison, autocratic political regimes are less conducive to such progress.

The relationship between democracy and innovation at the national level has attracted the attention of scholars with mixed findings. According to Coccia [8] "democracy richness" refers to the level of democratization in a country, measured with liberal, participatory, and constitutional democracy indices. The study establishes a positive relationship between democratization and technological innovation and gives several reasons. Firstly, the study suggests that democratization can lead to an increase in technological innovation because it creates a more open and competitive environment that encourages innovation. Secondly, the study suggests that democratization can lead to an increase in technological innovation because it allows for a wider range of people to participate in the innovation process, which can lead to more diverse and innovative ideas. Thirdly, the paper suggests that technological innovation can lead to an increase in democratization because it can create new economic opportunities and increase the standard of living, which can lead to demands for greater political freedom and democracy. Finally, the paper suggests that the relationship between democratization and technological innovation is complex and bidirectional, with each factor influencing the other in a feedback loop.

Coccia [48] suggests that the factors that contribute to technological innovation include efficient national systems of innovation, fruitful university-industry-government linkages, effective institutions based on higher levels of democracy, higher R&D spending by governments and business enterprises, active industrial structure and service sector, and high-skilled immigration inflows. Additionally, the study shows a positive relationship between innovative capacity and GDP per capita, which is a main determinant of the patterns of technological performance.

Coccia [49] argues that higher religious fractionalization, which is a proxy for cultural diversity, may support innovative outputs, particularly among richer and more democratic countries. The study analyzes the role of predominant religious cultures in various countries and their impact on technological innovation. The level of democratization in a country can also play a role in the impact of religious culture on technological innovation. Factors such as democratization and socio-economic determinants can influence patterns of technological innovation in different countries in several ways. Democratization can be a driving force for technological change and innovation. Countries with higher levels of democracy tend to have a higher level of technology than less free and more autocratic countries. This suggests that democratization can create an environment that is more conducive to technological innovation.

According to Coccia [50], religion shapes people’s attitude of mind, education, culture, and institutions of countries, and is likely a main socio-cultural determinant of the patterns of technological innovation However, religion is not the only factor that influences technological outputs of countries, as there are other intrinsic factors such a wealth and democratization of the society and economic system that also drive patterns of technological innovation.

In Gao, Zang [11], the researchers utilized a patent application database from the United States National Bureau of Economic Research and a democracy variable obtained from the Polity IV project. Their empirical analysis was carried out using cross-national panel data and conventional ordinary least squares methods with fixed effects. The research indicates that there is no statistically significant impact of democracy on innovation. However, it is noteworthy that despite serving as a gauge of technological advancement, a patent application is not without drawbacks. In the manufacturing sector innovation context, patent applications are often considered a reliable indicator. However, it is essential to note that patent applications may not provide a comprehensive representation of the entire country, potentially leading to biased outcomes.

Wang, Feng [12] use both patent and trademark applications from World Development Indicator to measure innovation and further scrutinize the influence of democracy on innovation. Using the system GMM method the study shows a positive relationship between democracy and innovation. The study argues that the applications of patents or trademarks show the progress of accession, and preceding performance plays a major role in their ongoing behavior.

## 3. Materials and methods

### 3.1. Sample and data

The study utilizes data on a sample of 61 developing countries from 2013 to 2020 making a strongly balanced panel. The middle- and low-income countries according to the definition of the World Bank are taken as developing countries [51]. The number countries and years of data are limited by the availability of data at the time of analysis. The list of the countries included in the analysis is given in the Appendix A in S1 Appendix.

### 3.2. Measure of variables

#### Innovation

The primary variable of interest to capture innovation in a country is the Innovation Output Index. The Innovation Output Index is a component of the Global Innovation Index (GII). In 2007 while working at INSEAD, Professor Dutta initiated the Global Innovation Index Project with the objective of identifying metrics and techniques that could provide a more comprehensive assessment of innovation in society, surpassing conventional measures like quality of research papers and the amount of investment in research and development [52].

The Global Innovation Index (GII) seeks to go beyond traditional measures of innovation such as the number of research articles and the level of research and development (R&D) expenditures. Instead, the GII aims to capture the richness of innovation in society by considering a broader and more horizontal definition of innovation, which includes social innovations, business model innovations, and technical innovations. This broader perspective reflects the evolving nature of innovation and the recognition that innovation is not solely restricted to R&D laboratories and published scientific papers.

The Global Innovation Index (GII) helps create an environment for continual evaluation of innovation factors by providing a rich database of detailed metrics for refining innovation policies. It serves as a key tool for assessing the climate and infrastructure for innovation and related outcomes, allowing for ongoing evaluation and refinement of innovation policies. Additionally, the GII is designed to move beyond the mere measurement of simple innovation metrics, integrating new variables and incorporating newly available data inspired by the latest research on the measurement of innovation. This approach enables the GII to contribute to the ongoing evaluation and improvement of innovation factors on a global scale.

The Global Innovation Index (GII) comprises of a duo of sub-indices. The Innovation Input Index and Innovation Output Index are two metrics used to measure innovation within a given context. The Innovation Input Index comprises five fundamental pillars: institutions, human capital and research, infrastructure, market sophistication, and business sophistication. The Innovation Output Index comprises two fundamental pillars: Knowledge and Technology Outputs and Creative Outputs. The Knowledge and Technology Outputs pillar is made up of three measures: Knowledge Creation, Knowledge Impact and Knowledge Diffusion. The Creative Outputs pillar is composed of three measures: Intangible Assets, Creative Goods & Services, and Online Creativity. These measures are further composed of several indicators, each making the Innovation Output Sub-index quite a comprehensive index. To see all the indicators of the Innovation Output Index, please refer to Appendix B in S1 Appendix.

According to Huarng and Yu [53] Global Innovation Index is a widely used measure of innovation and many studies have utilized it for analyzing national comparative innovation competence, see for example Wonglimpiyarat [54], Al-Sudairi and Haj Bakry [55], Sohn, Kim [56], Hamidi and Berrado [57]and Jankowska, Matysek-Jędrych [58].

#### Democracy

To effectively measure democracy, it is crucial to have indicators that capture democratic rights and the rule of law. According to Elkins [59] the empirical tests of alternative conceptualizations, have implications for our understanding of democracy. The evidence suggests that graded measures have superior validity and reliability, indicating that specific cases correspond to the concept of democracy to varying degrees, which can and should be measured. This challenges the insistence on dichotomous measures and widens an important division among scholars about the conceptualization of democracy. The empirical tests provide support for the meaningful variation in the degree of democracy across time and space and demonstrate that measures of democracy which provide for gradations best fit the behavior that theoretical work on democracy would predict. Therefore, the implications include a reevaluation of the measurement of democracy and a potential shift towards using graded measures to capture the nuances of democratic systems.

One approach to assessing democratic rights is through the utilization of freedom ratings by Freedom House, as highlighted by Alexander and Welzel [60]. These ratings offer comprehensive data that encompass the rights essential to liberal democracy [61], thereby providing valuable insights into the spatial and temporal aspects of democracy. Although the coding rules associated with the freedom ratings have faced criticism for their lack of transparency [62], it is worth noting that these ratings exhibit a high level of measurement reliability when compared to other democracy indices [63,64]. Consequently, the use of freedom ratings as an indicator of democratic rights can be justified.

The freedom ratings are divided into two indices: the ’civil liberties’ ratings primarily focus on private freedoms that signify autonomy rights, while the ’political rights’ ratings shed light on public freedoms that reflect participation rights. This classification allows for a comprehensive understanding of the various dimensions of democratic rights and enables a more nuanced analysis of the democratic landscape.

By incorporating the freedom ratings from Freedom House, researchers and scholars gain access to a robust measurement tool that facilitates comparative analysis and benchmarking of democratic progress. However, it is essential to acknowledge the ongoing discussions surrounding the transparency of coding rules and consider additional sources or indices to supplement the assessment of democracy.

Each country or territory is evaluated using a point-based system in the Freedom in the World Index, with a range of 0 to 4 points assigned to 10 political rights indicators and 15 civil liberties indicators. These indicators are in the form of questions, where a score of 0 represents minimal freedom, and 4 signifies the highest degree of freedom.

The political rights indicators have been classified into three distinct subcategories, namely Electoral Process, Political Pluralism and Participation, and Functioning of Government. The subcategory of the Electoral Process comprises three questions, while Political Pluralism and Participation consists of four questions. The Functioning of Government subcategory encompasses three questions. The civil liberties indicators have been categorized into four subcategories, namely Freedom of Expression and Belief (consisting of four questions), Associational and Organizational Rights (comprising of three questions), Rule of Law (comprising of four questions), and Personal Autonomy and Individual Rights (comprising of four questions). Additionally, the political rights section includes a discretionary question addressing forced demographic change. In this case, a score ranging from 1 to 4 may be subtracted, depending on the severity of the situation. The overall maximum score for political rights is 40 (4 points for each of the ten questions), while the maximum score for civil liberties is 60 (4 points for each of the 15 questions).

The scores from the previous edition serve as a benchmark for the current year’s assessment, with changes typically made only if significant real-world developments have occurred during the year, warranting a decline or improvement in the scores. The scores of political rights (PR) and civil liberties (CL) are added to calculate the overall Freedom in the World Index. The highest possible score for a country is 100, with a higher score indicating a greater degree of democracy within the country. The studies which have used Freedom house rating as measure of democracy in innovation related research include Coccia [8], Coccia [49], Coccia [65].

To check the robustness of estimates, we use an alternative but widely used measure of democracy called the Polity2 indicator of the Polity IV project of the Center of Systemic Peace. The polity measure of democracy has more than 5000 citations. Initially built upon Gurr [66] and Eckstein [67] research on political systems, the aim of this analysis was to examine whether there are discernible historical or cross-cultural trends in the commonly held beliefs regarding the characteristics of state authorities worldwide. The Polity index comprises two sub-indices, namely Institutional Democracy and Institutional Autocracy, which gauge the democratic and autocratic aspects of a nation, respectively. These sub-indices are derived from various specific sub-components and are ultimately combined to form the Polity index. Spanning a 21-point scale, ranging from more autocratic to more democratic, this index provides annual data since 1800 for all countries globally with a population exceeding 500,000 [68].

#### GDP per capita

One of the control variables considered in our analysis is the gross domestic product (GDP) per capita. While it is important to note that the causal relationship between GDP and innovation operates in both directions, with innovation being viewed as a critical determinant of long-term economic growth, several factors influence the impact of innovation on growth.

Empirical studies have revealed that the impact of innovation on growth is constrained by factors such as social capital [69], financial development, contract enforcement, supporting and complementary capacities [70], and other related factors. Additionally, increasing incomes have been found to stimulate innovation by generating demand for diverse and sophisticated consumer products.

Thus, it is imperative to incorporate GDP per capita as a covariate in our examination. The reason for this is that GDP per capita exhibits a correlation not only with innovation but also with other plausible factors that may influence innovation, including democracy, education, and urbanization. Omitting GDP per capita as a control variable could result in the introduction of omitted variable bias, thereby impacting the accuracy of our estimation regarding the impact of democracy on innovation. By including GDP per capita as a control variable, we can more accurately examine the relationship between democracy and innovation while accounting for the potential influence of economic factors [11].

#### Population density

Kremer [71] suggests that higher population density can stimulate innovation by fostering the implementation of novel ideas. It also promotes the adoption and diffusion of new technologies or products. Thus, we consider population density, measured as the number of people per square km (Density), as an explanatory variable to capture the accumulation and exchange of knowledge and novel ideas, as recommended by [12].

#### Trade openness

Importantly, international trade has the potential to generate spillover effects of advanced technologies and enhance a country’s absorptive capacity, thereby stimulating domestic innovation Gao, Zang [11]. Therefore, we incorporate the measure of economic openness, represented by the share of exports and imports concerning the Gross Domestic Product (GDP), to account for the impact of economic openness on innovation. This control variable allows us to consider how the degree of economic openness influences the innovation process [12].

### 3.3. Models and data analysis procedure

Panel regression is more effective than time series predictions for a variety of reasons, including the fact that it can manage possible issues such as missing data. Additionally, panel statistics provide greater information regarding the dynamic progression of an individual element. In conclusion, the two dimensions of individual and time of panel data increase the panel sample’s capacity, ultimately improving the estimations’ precision [72]. To conduct the empirical estimation, we also use panel data. Other innovation-related studies which use panel data include Wang, Feng [12], Wang, Feng [73], Zheng, Feng [74], and Gao, Zang [11].

It makes sense, and also, past studies have demonstrated that innovation is dependent not just on the present economic climate but also on earlier technological advancement. In other terms, innovation is a dynamic process of evolutionary nature [12]. Hence, we employ the dynamic panel model and generalized method of moments (GMM) estimates to examine the effect of democracy on innovation.

System GMM is a statistical method used in panel data analysis to estimate the parameters of dynamic panel data models. This method is particularly useful when dealing with endogeneity issues in panel data, such as reverse causality and omitted variable bias. In system GMM, the estimation is done by using moment conditions that are based on lagged values of the dependent variable and the exogenous variables. The method involves two steps: the first step uses the lagged levels of the dependent variable and exogenous variables as instruments to estimate the parameters, while the second step uses the lagged first differences of the dependent variable and exogenous variables as additional instruments to improve the efficiency of the estimates. System GMM is considered a powerful method because it is robust to different forms of heteroscedasticity and serial correlation in the error term. It can also handle unobserved individual heterogeneity and endogenous variables [75,76].

Overall, System GMM is a flexible and effective method for analyzing dynamic panel data, particularly in the presence of endogeneity issues, and it is widely used in economics, finance, and other social sciences.

**Fig 1:**
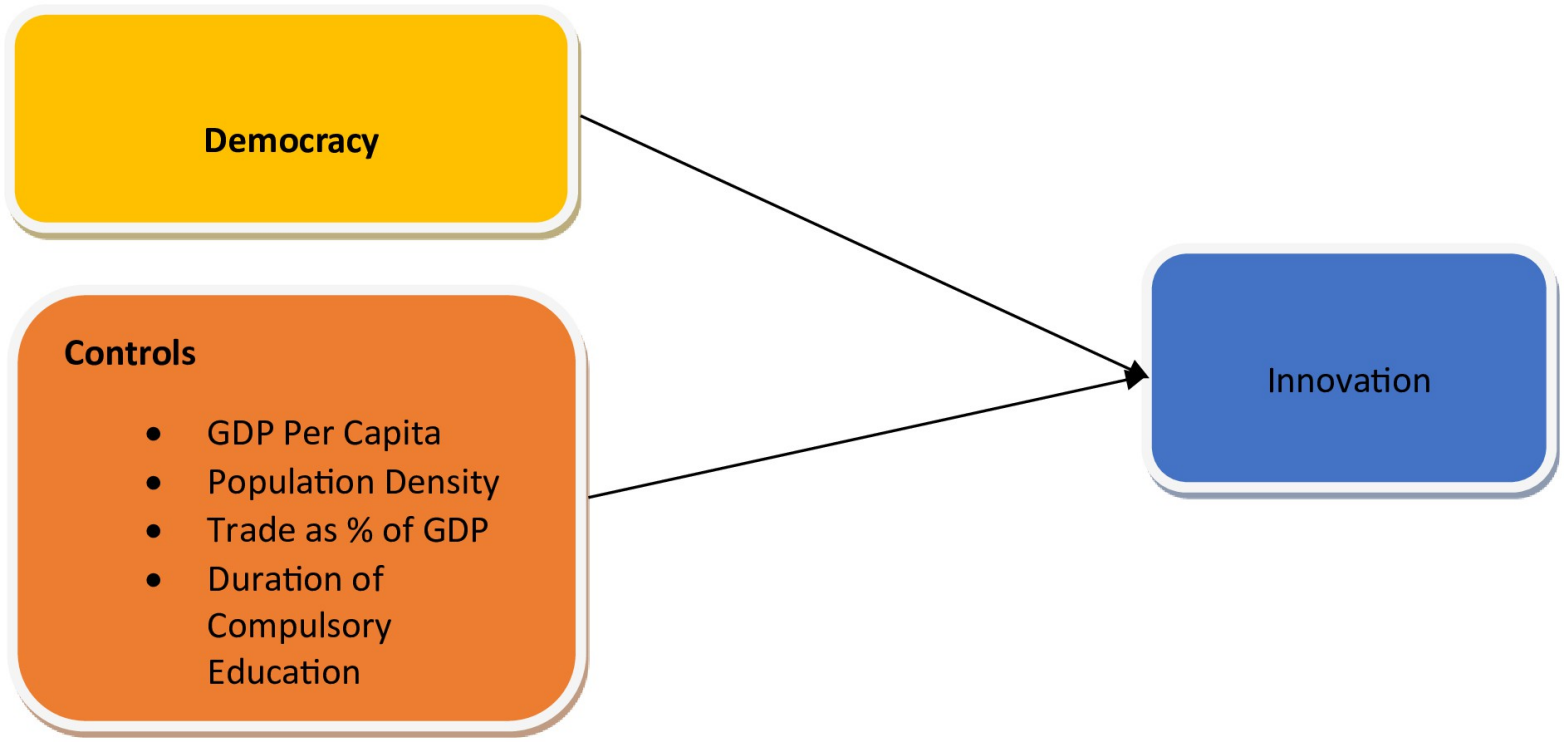
Theoretical framework linking independent variables (left) with dependent variable (right). Our model is specified as follows.

The theoretical framework showing the relationship between independent and dependent variables is given in Fig 1.

$${\text{I}}_{\text{it}}=\alpha +{\beta \text{D}}_{\text{it}}+\lambda \text{X}+\mu_{\text{t}}+\varepsilon_{\text{it}}$$

In this context, the variable I_it_ represents technical innovation, D_it_ denotes democracy, and X is a collection of other explanatory variables. The individual and time-fixed effects are measured by u_i_ and u_t_, respectively. The error term is ε_it_, and i represents the individual, where i = 1, 2, 3…N, while t represents the year, where t = 1, 2, 3…T. Furthermore, the inclusion of lagged dependent variables in the estimations may effectively address concerns about the selection of instrument variables and potential endogeneity. The GMM model incorporates the lag term of innovation in its construction to attain the desired outcome.

## 4. Results and discussion

Table 1 shows the descriptive statistics. The baseline regression results are shown in Table 2. The first three columns show the impact of democracy measured by the Freedom House Index (FHI) on the Innovation Output Index (IOI) using the OLS regression method. The results show no significant impact of democracy on innovation. However, since this is an OLS model that does not control for fixed effects and endogeneity, we cannot confidently say that this result gives us a causal relationship. To control for fixed effects and endogeneity, we use fixed effects and System GMM regression methods. The estimated results are reported in column (3) and column (4) of Table 2. We find that the democracy index is insignificantly associated with the innovation output index.

**Table 1:** Summary statistics.

	Mean	Median	Std. Dev.	Skewness	Kurtosis
Innovation Output Index	22.59	21.7	7.55	.66	4.31
Freedom House Index	51.48	52	22.02	.04	2.2
Polity2	3.85	6	5.5	-.83	2.27
Political Rights	20.57	22	10.47	-.1	2
Civil Liberties	30.91	30.5	11.92	.16	2.44
GDP Per Capita	4718.62	3867.82	4090.9	1.25	4.11
Population Density	129.25	76.34	188.14	3.74	20.09
Trade as % of GDP	66.5	60.31	28.11	.67	2.66
Duration of Compulsory Education	10.31	10	2.56	.13	2.5

**Table 2:** Regression results with Freedom House Index as measure of democracy.

Dependent Variable	(1)	(2)	(3)
*Innovation Output Index*	OLS	Fixed Effects	SystemGMM
Freedom House Index	-.0341	-.0677	.0705
	(.0256)	(.0562)	(.087)
GDP Per Capita	.0012***	.002***	.0004
	(.0002)	(.0007)	(.001)
Population Density	.0016	-.0038	.0303
	(.0041)	(.0219)	(.0187)
Trade as % of GDP	.0045	-.0025	.0642
	(.0163)	(.032)	(.0453)
Duration of Compulsory Education	-.1052	-.0802	-.9172
	(.2692)	(.5733)	(.9539)
L.Innovation Output Index			.8794***
			(.0799)
Constant	24.7316***	23.3406***	-3.3827
	(3.2311)	(7.7919)	(6.62)
Observations	438	438	382
R-sq	.6041	.6115	-
F-stat	-	27.6006	-
Adj R^2^	-	.6005	-

Standard errors are in parentheses.

*** p < .01,

** p < .05,

* p < .1.

The Freedom House Index (FHI) is comprised of two components, i.e., Political Rights (PR) and Civil Liberties (CL). In recent political economy and political science literature, there is widespread acceptance that political rights play a crucial role in democracy. These rights are commonly defined based on the idea of ensuring free and fair elections. More specifically, they encompass the establishment of an electoral process characterized by these qualities at various levels, including the executive, legislative, and local or regional levels. Another important aspect is creating an atmosphere free from intimidation and coercion, allowing citizens to participate openly and extensively as voters, candidates, and members of political parties. Additionally, these rights entail the implementation of mechanisms that connect the policies implemented by elected leaders to their scrutiny in a transparent manner, leading to accountability [77].

In terms of safeguarding individual rights, civil liberties are generally acknowledged as a fundamental component of democracy. For the past two centuries, individual rights have gained recognition as indispensable attributes of democracy and have been enshrined in the constitutions of many nations. These individual rights, often referred to as first-generation human rights, typically encompass freedom of speech, freedom of assembly, and a category that is challenging to define precisely. This category is occasionally described as due process protection, equal treatment under the law, or protection against arbitrary actions by the state [77].

In the next step, we check the individual impact of both of these components of democracy on innovation. The results are shown in Tables 3 and 4. The results show that neither political rights nor civil liberties have any significant impact on innovation outcomes. All three models, i.e., OLS, Fixed Effects, and System GMM, give similar results. Results are displayed in Tables 5 and 6 given in the Appendix C in S1 Appendix.

**Table 3:** Regression results with Polity2 as measure of democracy.

Dependent Variable	(1)	(2)	(3)
*Innovation Output Index*	OLS	Fixed Effects	System GMM
Polity2	-.0361	-.179	-.2801
	(.104)	(.1238)	(.2047)
GDP Per Capita	.001***	.0025**	.0005
	(.0002)	(.0011)	(.0008)
Population Density	-.0003	-.0121	.009
	(.0041)	(.0302)	(.0196)
Trade as % of GDP	.0034	-.0154	.0198
	(.0192)	(.0453)	(.0389)
Duration of Compulsory Education	-.312	-.5895	-.8124
	(.2822)	(.4117)	(.5044)
L.Innovation Output Index			.7278***
			(.0973)
Constant	26.1208***	25.3977***	9.8066
	(3.3662)	(6.7035)	(8.7948)
Observations	322	322	267
R-sq	.4715	.4884	-
F-stat	-	16.136	-
Adj R^2^	-	.4719	-

Standard errors are in parentheses.

*** p < .01,

** p < .05,

* p < .1.

**Table 4:** Synthesized results.

Measure of Democracy	Impact on Innovation		
	OLS	Fixed Effects	SystemGMM
Freedom House Index	No significant impact	No significant impact	No significant impact
Polity2 Indicator	No significant impact	No significant impact	No significant impact

To check the robustness of estimates, we use an alternative but widely used measure of democracy called the Polity2 indicator of the Polity IV project of the Center of Systemic Peace.

Table 3 shows the results when we use the Polity2 indicator as a measure of democracy. The results are similar to those obtained when we use the Freedom House Index (FHI) to measure democracy. There appears to be no significant impact of democracy on innovation. The results are synthesized in Table 4 below.

We can find support for our results from the literature. A recent study by Gao, Zang [11] have shown that there is no significant impact of democracy on innovation. The study argues that Governments in autocratic nations may encounter fewer obstacles than their democratic counterparts when it comes to allocating more excellent resources toward scientific and technological research, including specialized areas such as military and defense, as exemplified by Russia and China.

Previous research has indicated that the decentralized approach to innovation, which involves private investment in research and development (R&D) and is typically associated with democratic systems, often leads to insufficient investment [78]. Additionally, empirical evidence suggests that the early phases of the shift from an autocratic, socialist framework to a democratic, capitalist one may adversely impact the sustainability of even long-standing and technologically sophisticated corporations [79]. These findings align with our study, as they emphasize the risks associated with democratic transitions concerning innovation and shed light on the potential disadvantages of R&D models in democratic nations.

To be effective democracy takes a long time. Modelski and Perry [80] argue that democratization consolidation process takes approximately 228 years for going from 10% to 90% democratization. The implication for the future is that democratization is not a smooth process but moves in discrete increments or waves [81]. Basic electoral democracies for instance may have a long way to go before graduating to full liberal democratic status. We can safely assume that many democratic developing countries are relatively young democracies or just transitioning democracies [82]. This can help us in explaining that long established liberal democracies of North American and Western Europe are much more innovative than rest of the democratic and non-democratic countries.

The statistical evidence presented in Coccia [50] suggests that a higher religious fractionalization, which is a main proxy of cultural diversity, has a positive effect on technological outputs in advanced economies This relationship appears to be particularly true among richer and more democratic countries, which are mainly located in the European and North American geo-economic areas. However, the relationship between religious fractionalization and technological outputs is also driven by omitted factors influencing both socio-economic structure of countries and patterns of technological innovation. The high immigration rate to European and North American countries increases the cultural and religious diversity of those nations. Religious and cultural diversity plays an important role in technological innovation in richer democratic countries [49]. In the absence of religious and cultural diversity democracy may not be able to influence innovation significantly.

Some scholars like Inglehart and Welzel [83] differentiate between institutional and effective democracy. According to the book focusing on freedom and self-expression are more important to democracy than is overt support for democratic institutions. This is true because democracy does not reflect a merely institutional phenomenon. It reflects a civic phenomenon, involving citizens who practice democratic principles in their daily lives. This confirms that making democracy work requires civic values among the public. Regime type may also matter for democracy to be effective as pointed out by Norris [84]. She finds that it is the power-sharing rather than the power-concentrating versions of these institutions e.g., proportional representation electoral systems rather than majoritarian systems, federal rather than unitary states that are associated with higher levels of democracy. Further research linking regime type to innovation is required to shed more light on this aspect.

Finally democracy may not have any significant impact on innovation in the absence of other factors. Socio-economic determinants such as economic governance, demographic change, social and cultural openness, national and regional systems of innovation, and the rule of law can also influence patterns of technological innovation. For example, a country with a higher GDP per capita may have more resources to invest in research and development, which can lead to higher levels of technological innovation. Institutions such as patent offices can also play a role in patterns of technological innovation. Countries with better institutions tend to have better support for patterns of technological innovation [49]. Democracies like India lack in rule of law, property rights enforcement [85] and other necessary institutional prerequisites required for attracting individuals and organizations to invest to innovate.

## 5. Conclusion

In this study, our objective was to examine the causal relationship between democracy and innovation in developing countries. Our analysis reveals that the impact of democracy on innovation is not statistically significant. Thus, the empirical evidence presented in our panel data models does not support Popper’s hypothesis in developing countries, which we initially introduced in our discussion. Importantly, these findings remain consistent across three different regression models and alternative democracy indexes, confirming the robustness of our results.

Nevertheless, it is essential to exercise caution when interpreting our results due to a few critical considerations. Firstly, despite our findings, there exist qualitative historical studies that focus on the relationship between innovation and democracy [11,48–50,86], as well as innovation and specific policies [87–89]. According to the findings of these studies, the implementation of liberal policies by democratic governments can have a substantial positive impact on innovation achievement. It is important to acknowledge that a direct comparison between our findings and those of previous studies is not feasible due to the utilization of a shorter time frame and a multi-country approach in our analysis.

While our research did not reveal a direct positive correlation between democracy and innovation, it is important to clarify that our intention was not to question the inherent value of a democratic political system or its potential positive impact on economic development. Additionally, further investigation is warranted to explore the indirect effects of democracy on innovation. Democratic countries tend to be more open in various aspects compared to autocratic countries. Even if they may not be at the forefront of innovation, democratic countries have a greater capacity to receive technology transfers from nations with similar political systems [90]. In the current era of globalization, it may be more efficient to pursue economic growth by importing advanced technologies from foreign sources, rather than exclusively prioritizing domestic innovation at any cost.

As pointed out by Gao, Zang [11], Innovation is deeply intertwined with social institutions and cultures. The mechanisms that facilitate innovation within a country require sufficient time to adapt to political changes. For instance, transitioning from a centrally driven innovation regime to one where individual firms play a leading role can be a lengthy process. It is possible that the adjustments needed in democratizing countries, which we examined in our study, may require more time than initially anticipated. The process of democratic transition and consolidation typically spans over two decades, and this timeframe may not provide ample opportunity for institutional reforms to firmly establish themselves and effectively stimulate innovation.

This study also has limitations. There are limitations of the data set approach to measuring democracy. There are several drawbacks, including the reliance on institutions and procedures as surrogates for substantive democracy, the reduction of rich qualitative data to a three-way categorization of countries based largely on the existence of democratic procedures and institutions, and the trade-offs between intensive and extensive research strategies. Additionally, some data sets may not cover all countries or have limited temporal coverage and democracy diffusion takes a long time. Furthermore, the over-reliance on tangible and transparent features of democratic systems, such as elections, constrains the discourse of democracy and limits the ability to adequately grasp the complex interaction between democratization and specific geographical-historical contexts. This suggests that the data set approach may overlook the nuanced and context-specific nature of democratization processes [91]. Freedom House and Polity ratings suffer from the same criticism.

Global Innovation Index (GII) is also not free of criticisms. Some studies have questioned the efficacy of the GII in measuring innovation and have raised concerns about its components and their impact on the rankings of countries [92]. Additionally, there are discussions about the limitations of the GII in capturing certain aspects of innovation, such as measuring creative outputs and the subjective nature of the data array over countries [93]. Some scholars have raised concerns about the methodology used in constructing the GII, suggesting that it may not adequately account for the complex and multifaceted nature of innovation systems in different countries. The GII heavily relies on quantitative metrics, which may not fully capture qualitative aspects of innovation, such as the quality of innovation, social impact, and inclusivity [94]. These criticisms highlight the need for a nuanced understanding of the GII and its components when assessing innovation potential. Also Critics argue that the GII may not fully capture the innovation activities and potential of all countries, especially those with different economic and social structures [56].

This study does not take into account the socio-cultural and religious factors which may work in conjunction with democracy to have a positive impact on innovation [49,50]. This study also does not take into account the level of consolidation of democracy in the countries under analysis nor it considers the variations in democracy and types of regimes [80,84,95] adding to the manifold limitations of the study.

The findings presented in this study underscore several key recommendations vital for boosting innovation in developing countries. These recommendations aim to influence future policymaking in this field. Through analysis of data and discussions conducted in preceding sections the following recommendations emerge as essential pathways for encouraging innovation in developing countries.

Firstly, policy makers should propose best practices directed to support a higher economic freedom in society, effective regulation, higher economic and political stability, good economic governance, and a higher level of education system. These are main preconditions for the origin, diffusion, and utilization of technology and economic growth within and between economic systems. Therefore, the political economy of growth should be designed considering the joint coevolution of democratic and social systems in order to support a fruitful institutional change and good economic governance for technical change directed to distribute total wealth among the widest fraction of population [65].

Secondly democracy in the absence of other socio-cultural and religious factors will not have a significant impact on boosting innovation. Studies have shown that the cultural and religious diversity play a significant role in promoting innovation [49,50]. This helps explain the elevated level of innovation in diverse immigrant-based societies like the USA and the UK. Therefore, policymakers in developing countries should actively promote cultural and religious diversity in their countries. Ethnic and religious minorities should be given equal access to education and capital resources. Policies may be enacted to attract talent and capital from other countries to increase innovation.

Finally, democracy takes a very long time to diffuse and consolidate. Therefore, policymakers in developing countries should remain committed to democracy and it will eventually bring its fruits. Patterns of stability, innovation and economic growth also depend on the nature of the political system in a country. Research has shown that consensus based proportional representation system may be better than majoritarian political system and federalism due to its power sharing arrangements may be preferable to unitary systems [84,95]. Policymakers may reevaluate the type of system prevalent in their respective countries. Which system suits any specific developing country needs to be carefully evaluated and reforms initiated in that direction.

## Supporting information

S1 Appendix (DOCX)
