# Supplementary material for: Democracy’s limited impact on innovation: Panel data evidence from developing countries
Source: PLoS One. 2024 Mar 15;19(3):e0297915. doi: 10.1371/journal.pone.0297915 (PMC10942087; doi:10.1371/journal.pone.0297915)
Supplement: S1 Appendix — (DOCX) [file pone.0297915.s001.docx]

**Appendix A**

**List of countries included in the analysis.**

| No. | Country Name | No. | Country Name |
| --- | --- | --- | --- |
| 1 | Albania | 32 | Malawi |
| 2 | Algeria | 33 | Malaysia |
| 3 | Argentina | 34 | Mali |
| 4 | Armenia | 35 | Mexico |
| 5 | Azerbaijan | 36 | Mongolia |
| 6 | Bangladesh | 37 | Montenegro |
| 7 | Belarus | 38 | Morocco |
| 8 | Bolivia | 39 | Mozambique |
| 9 | Bosnia and Herzegovina | 40 | Nepal |
| 10 | Botswana | 41 | Niger |
| 11 | Brazil | 42 | Nigeria |
| 12 | Cambodia | 43 | Oman |
| 13 | Chile | 44 | Pakistan |
| 14 | China | 45 | Paraguay |
| 15 | Colombia | 46 | Peru |
| 16 | Costa Rica | 47 | Philippines |
| 17 | Ecuador | 48 | Rwanda |
| 18 | Egypt | 49 | Senegal |
| 19 | El Salvador | 50 | South Africa |
| 20 | Ethiopia | 51 | Sri Lanka |
| 21 | Ghana | 52 | Tajikistan |
| 22 | Guatemala | 53 | Thailand |
| 23 | Honduras | 54 | Togo |
| 24 | India | 55 | Tunisia |
| 25 | Iran (Islamic Republic of) | 56 | Turkey |
| 26 | Jordan | 57 | Uganda |
| 27 | Kazakhstan | 58 | Uruguay |
| 28 | Kenya | 59 | Yemen |
| 29 | Kyrgyzstan | 60 | Zambia |
| 30 | Lebanon | 61 | Zimbabwe |
| 31 | Madagascar |  |  |

**Appendix B**

The Innovation Output Sub-Index Indicators

| Knowledge & technology outputs pillar | Creative outputs pillar |
| --- | --- |
| Knowledge and technology outputs  1 Knowledge creation  1.1 Patents by origin/bn PPP$ GDP  1.2 PCT patent applications/bn PPP$ GDP  1.3 Utility models by origin/bn PPP$ GDP  1.4 Scientific & technical articles/bn PPP$ GDP  1.5 Citable documents H index  2 Knowledge impact  2.1 Growth rate of PPP$ GDP/worker, %  2.2 New businesses/th pop.  2.3 Computer software spending, % GDP  2.4 ISO 9001 quality certificates/bn PPP$ GDP  2.5 High- & medium-high-tech manufactures, %  3 Knowledge diffusion  3.1 Intellectual property receipts, % total trade  3.2 High-tech exports less re-exports, % total trade  3.3 ICT services exports, % total trade  3.4 FDI net outflows, % GDP | Creative outputs  1 Intangible assets  1.1 Trademarks by origin/bn PPP$ GDP  1.2 Industrial designs by origin/bn PPP$ GDP  1.3 ICTs & business model creation  1.4 ICTs & organizational model creation  2 Creative goods and services  2.1 Cultural & creative services exp., % total trade  2.2 National feature films/mn pop  2.3 Global ent. & media market/th pop  2.4 Printing & publishing manufactures, %  2.5 Creative goods exports, % total trade  3 Online creativity  3.1 Generic TLDs/th pop  3.2 Country-code TLDs/th pop  3.3 Wikipedia monthly edits/mn pop  3.4 Video uploads on YouTube/pop |

Source: https://www.wipo.int/edocs/pubdocs/en/wipo_pub_gii_2016-annex1.pdf

**Appendix C**

**Table 5: Regression results impact of political rights on innovation**

|  | (1) | (2) | (3) |
| --- | --- | --- | --- |
| Dependent Variable  Innovation Output Index | OLS | Fixed Effects | SystemGMM |
| Political Rights | -.0485 | -.0811 | .1587 |
|  | (.0483) | (.0993) | (.1563) |
| GDP Per Capita | .0011*** | .0021*** | .0005 |
|  | (.0002) | (.0007) | (.001) |
| Population Density | .0017 | -.0019 | .0309* |
|  | (.0041) | (.0221) | (.0183) |
| Trade as % of GDP | .0054 | -.0001 | .0622 |
|  | (.0163) | (.0319) | (.0468) |
| Duration of Compulsory Education | -.1236 | -.0629 | -.966 |
|  | (.2701) | (.5862) | (.9403) |
| L.Innovation Output Index |  |  | .8853*** |
|  |  |  | (.0773) |
| Constant | 24.1339*** | 20.6637*** | -2.9426 |
|  | (3.1826) | (7.6969) | (5.8821) |
| Observations | 438 | 438 | 382 |
| R-sq | .6024 | .6095 | - |
| F-stat | - | 28.288 | - |
| Adj R^2^ | - | .5984 | - |
| *Standard errors are in parentheses* | | | |
| **** p<.01, ** p<.05, * p<.1* | | | |
|  | | | |

**Table 6: Regression results impact of civil liberties on innovation**

|  | (1) | (2) | (3) |
| --- | --- | --- | --- |
| Dependent Variable  *Innovation Output Index* | OLS | Fixed Effects | SystemGMM |
| Civil Liberties | -.0746 | -.1605 | .1025 |
|  | (.0488) | (.1107) | (.1629) |
| GDP Per Capita | .0012*** | .002*** | .0004 |
|  | (.0002) | (.0007) | (.001) |
| Population Density | .0015 | -.005 | .03 |
|  | (.0041) | (.0222) | (.0188) |
| Trade as % of GDP | .0031 | -.0071 | .063 |
|  | (.0163) | (.0326) | (.0448) |
| Duration of Compulsory Education | -.1093 | -.1334 | -.8373 |
|  | (.2666) | (.5646) | (.9437) |
| L.Innovation Output Index |  |  | .8769*** |
|  |  |  | (.0794) |
| Constant | 25.3838*** | 26.033*** | -3.4581 |
|  | (3.314) | (8.054) | (6.7062) |
| Observations | 438 | 438 | 382 |
| R-sq | .6055 | .6131 | - |
| F-stat | - | 26.4808 | - |
| Adj R^2^ | - | .6022 | - |
| *Standard errors are in parentheses* | | | |
| **** p<.01, ** p<.05, * p<.1* | | | |
|  | | | |
